# RCMV increases intimal hyperplasia by inducing inflammation, MCP-1 expression and recruitment of adventitial cells to intima

**DOI:** 10.1186/2042-4280-1-7

**Published:** 2010-12-23

**Authors:** Monika K Grudzinska, Krzysztof Bojakowski, Joanna Soin, Frank Stassen, Cecilia Söderberg-Nauclér, Piotr Religa

**Affiliations:** 1 Experimental Cardiovascular Research Unit, Department of Medicine, Solna, Karolinska Institutet, Stockholm, Sweden; 2 Department of General, Vascular and Oncologic Surgery, Medical University of Warsaw, Warsaw, Poland; 3 Department of General Biochemistry and Nutrition, Medical University of Warsaw, Warsaw, Poland; 4 Maastricht University Medical Center, Maastricht, The Netherlands

## Abstract

**Background:**

Cytomegalovirus (CMV) infection has been associated with accelerated transplant vasculopathy. In this study, we assessed the effects of acute rat CMV (RCMV) infection on vessel remodeling in transplant vasculopathy, focusing on allograft morphology, inflammation and contribution of adventitial cells to intimal hyperplasia.

**Methods:**

Infrarenal aorta was locally infected with RCMV and transplanted from female F344 rats to male Lewis rats. Graft samples were collected 2 and 8 weeks after transplantation and analyzed for intimal hyperplasia, collagen degradation and inflammation. Transplantation of aorta followed by transplantation of RCMV infected and labeled isogenic adventitia were performed to study migration of adventitial cells towards the intima.

**Results:**

Intimal hyperplasia was increased threefold in infected allografts. RCMV induced apoptosis in the media, expression of matrix metalloproteinase 2, and decreased collagen deposits. Macrophage infiltration was increased in the infected allografts and resulted in increased production of MCP-1. RCMV-infected macrophages were observed in the adventitia and intima. Cells derived from infected adventitia migrated towards the intima of the allograft.

**Conclusions:**

RCMV enhances infiltration of macrophages to the allografts, and thereby increases MCP-1 production and inflammation, followed by recruitment of adventitial cells to the intima and accelerated intimal hyperplasia.

## Background

Cytomegalovirus (CMV) is a well-known risk factor for late allograft dysfunction and is also associated with atherosclerosis and restenosis after angioplasty [1-6]. The major cause of late organ dysfunction after transplantation is accelerated transplant vasculopathy (TV), characterized by diffuse concentric intimal proliferation that results in vessel occlusion.

In the early phase of TV, injured vessels contain predominantly macrophages and a few subendothelial lymphocytes (T, B, and natural killer cells); late vascular lesions are associated with a thickened intima containing cells of smooth muscle cell (SMC) phenotype interspersed with macrophages [7]. Intimal lesions are thought to be populated by dedifferentiated SMCs or vascular progenitor cells, either circulating or resident in the vessel wall, e.g. in the adventitia [8]. Furthermore, evidence has recently emerged suggesting that a vascular adventitia is activated in a variety of vascular diseases and plays an important role in the progression of vascular inflammation [9,10]. The number of progenitor cells contributing to vascular remodeling is thought to increase with the inflammatory response and their migration to sites of vascular injury is mediated by factors such as monocyte chemoattractant protein 1 (MCP-1) [11].

Clinical data and animal studies suggest that CMV contributes to the development of TV [12]. CMV infection of macrophages and endothelial cells affects cellular processes that may contribute directly to vascular disease. For example, CMV antigens activate the immune system [13] and hence may drive an inflammatory process in the arterial wall [14-17]. It is hypothesized that a local injury (e.g., allospecific injury or balloon angioplasty) can reactivate latent CMV in cells in the vessel wall or in cells recruited to the site of injury [2,18-20]. The virus then initiates an acute infection and inflammation, which may affect the migration and proliferation of vascular cells. In vitro, human CMV (HCMV) infection mediates vascular SMC migration that is dependent on the expression of the virally encoded chemokine receptor homologue US28 [21]. Deletion of the US28 rat homolgue R33 reduces the capacity of CMV to accelerate chronic rejection and TV in a rat model [22].

The mechanisms of HCMV-associated TV are difficult to determine because of its multifactorial etiology. Moreover, HCMV is ubiquitous and infects multiple cell types, including SMCs, endothelial cells, macrophages, and fibroblasts, and a primary infection is followed by lifelong latency, which makes it difficult to establish a specific temporal relationship between infection and TV [5]. Therefore, animal models are ideal for studying the association between CMV and TV.

The rat cytomegalovirus (RCMV) model has been useful to study mechanisms of CMV-related diseases, including the effect of the virus on TV [5,22-27]. In immunocompromised rats, RCMV causes a widespread infection of most tissues and various cell types, including endothelial cells, epithelial cells, macrophages, polymorphonuclear cells, and fibroblasts [28]. However, the effects of CMV infection on TV in rats and mice have so far only been studied after systemic infection [5,23-25,29-32].

To investigate the impact of a local CMV infection on cellular activation and vascular graft morphology in TV, we used a rat model in which the aorta or adventitia were locally infected with RCMV *ex vivo *after collection from the donor rat and before transplantation into the recipient rat. This model aims to mimic the effects of a severe systemic infection on the allograft and gives insight into which cells in the allograft become infected, and enable investigation of the cellular immune response against the virus and its impact on vascular remodelling.

## Methods

### Ethics statement

All animal procedures were approved by ethical committees of Stockholm North Ethical Committee and performed in accordance with institutional guidelines and conformed to the Guide for the Care and Use of Laboratory Animals at Karolinska Institute, Stockholm, Sweden.

### RCMV propagation

RC 127 (Maastricht strain, 2.1 × 10^6 ^pfu/ml; multiplicity of infection, 3.5 × 10^-2^) was originally isolated from wild rats [33]. RCMV was propagated by infecting fibroblasts prepared from 17-day-old DA rat embryos. The supernatant was used for in vivo infection. Fibroblasts were cultured in flasks containing modified Eagle's minimum essential medium (Flow Laboratories) supplemented with 200 mmol/l L-glutamine (Northumbria Biologicals), antibiotic solution (10,000 IU/ml penicillin and 1000/ig/ml streptomycin) (Gibco), and 10% FCS (Sera-Lab) at 37°C in a 5% CO_2 _incubator. At confluency, the cells were infected with RCMV according to standard viral culture techniques [34] and maintained in culture medium (as above) supplemented with 2% FCS (Sera-Lab). After 5 to 7 days, when a cytopathic effect was observed in at least 95% of the cells, the cells were detached from the bottom of the flask by tapping and thereafter centrifuged at 1200 rpm for 5 min. The supernatant containing free RCMV was collected and immediately stored at -70°C. The virus was a gift from Prof. Cathrien A. Bruggeman's laboratory.

### Animal transplant models of locally infected with RCMV aorta and adventitia and study design

Inbred male (150-170 g) Lewis rats (LEW.RT1 strain) and female Fisher rats (F344.RT1v1 strain) were used.

Rats used for aorta transplantation study (n = 96) were divided into two groups: allograft (F344 to LEW) and isograft (LEW to LEW). The allograft group was divided into two experimental groups (n = 12 rats each): (1) RCMV-infected allografts collected after 2 weeks (6 grafts); (2) RCMV-infected allografts collected after 8 weeks (6 grafts); and two control groups (n = 12 rats each), (3) uninfected allografts collected after 2 weeks (6 grafts), and (4) uninfected allografts collected after 8 weeks (6 grafts). Uninfected and infected isogenic grafts, isografts, (n = 12 rats in each group) were used as controls and were included to certify that there was no intimal hyperplasia in the isografts as was previously reported [27,31,35]. Transplantations of the infrarenal rat aorta: allograft (F344/LEW rats) and isograft (LEW/LEW rats) were performed as described [36]. A part of the infrarenal aorta was taken from the donor, incubated for 20 min with 2.1 × 10^6 ^pfu RCMV (tissue culture-derived virus diluted in 1 ml of PBS), and transplanted into recipient rats. Allograft and isograft samples were collected 2 and 8 weeks post transplantation.

Thirty-six rats were used for adventitia transplantation. 12 rats (F344) served as donors of aorta, 12 rats of LEW strain served as donors of adventitia, and 12 rats of LEW strain served as recipients of both aorta and adventitia. Four weeks after aortic abdominal allograft transplantation (F344 to LEW), isogenic adventitial cells (LEW to LEW) labeled with cell tracker, which does not leak to adjacent cells (Molecular Probes, Carlsbad, CA), were transplanted to the allograft by cuffing labeled adventitia around the previously transplanted allograft. Allograft samples were collected 2 weeks post adventitia transplantation and studied using confocal microscopy for the migration of adventitial cells to the intima *in vivo*.

### Histologic and morphometric analyses of the allografts

Transplanted aortas were collected, rinsed with 0.9% sodium chloride, fixed in 3% buffered formaldehyde for 4 h, and stored at 4°C in PBS with 0.02% sodium azide. Thereafter, paraffin blocks were made and allografts were cut to obtain transverse sections at 5 μm. The sections were stained with hematoxylin and examined with a Leica light microscope. Intima and media cross-sectional areas were measured with LeicaQWin software. The analysis was performed in a blinded approach by two observers who were unaware of coding.

### Immunohistochemistry of cross sections of the allografts

The following primary antibodies were used for immunohistochemistry: mouse anti-human SM α-actin, which is known to stain rat SM α-actin [37] (Dako), mouse anti-rat CD45 (BD Pharmingen), mouse anti-rat CD68 (Serotec), mouse anti-rat CD3 (GeneTex), mouse anti-rat caspase 3 (Neomarkers), mouse monoclonal antibodies against MMP-2 and MMP-9 (Abcam), mouse anti-rat MAb8 against RCMV early/late antigen (R44-encoded protein, gift from C. Bruggeman), and mouse anti-MCP-1 (Biolegend). Briefly, 5-μm paraffin sections were deparaffinized, hydrated, and digested with pepsin. The sections were then subjected to antigen retrieval in citrate buffer followed by peroxidase (Innovex Sciences), avidin, and biotin (Dako) and Fc receptor blockage (Innovex Sciences). Sections were incubated with monoclonal antibodies, and the signal was visualized with a horseradish peroxidase detection system (BioGenex) using diaminobenzidine (Innovex Sciences) as the chromogen. For double immunohistochemistry, the signal was detected with Vector alkaline phosphatase using BCIP/NBT as substrate. After counterstaining with hematoxylin (Sigma-Aldrich), specimens were mounted in permanent mounting medium (Histolab).

### Masson's trichrome staining of cross sections of the allografts

RCMV-infected and uninfected rat allografts collected at 2 and 8 weeks after transplantation were stained with Masson's trichrome stain to identify collagen deposits in the media as described [38]. Collagen deposits were assessed by computer-assisted histomorphometric image analysis in Adobe Photoshop. To estimate collagen content in the specimen, blue-stained areas were selected and quantified by an image-analysis system. The ranking procedure was performed in a blinded approach by two observers who were unaware of the coding.

### Statistics and data analysis

The analysis was done on the cross sections of the allografts. The numbers of macrophages, lymphocytes, SMCs and apoptotic SMCs among all intimal and medial cells were determined by manual counting of cells positive for CD68, CD45, CD3, SM-α actin and cells double positive for caspase 3/SM-α-actin. RCMV-infected macrophages were quantified by calculating the percentage of cells double positive for late/early RCMV R44 protein and CD68 among all CD68-positive cells. MMP content was determined as the area immunostained, expressed as a percentage of the total area. Collagen deposits (blue-stained areas) and MCP-1 content (green-stained areas) were assessed by computer-assisted histomorphometric image analysis in Adobe Photoshop. All ranking procedures were performed in a blinded approach by two observers who were unaware of coding. Results in multiple groups were analyzed with a two-tailed t-test, where the sample mean of the data obtained from infected allografts was compared to uninfected ones (2 weeks infected vs. 2 weeks uninfected, and 8 weeks infected vs. 8 weeks uninfected). P value < 0.05 was considered as significant.

## Results

### RCMV infection of aortic allografts enhances intimal hyperplasia and media destruction, and decreases extracellular matrix deposition in the allograft

**Figure 1:**
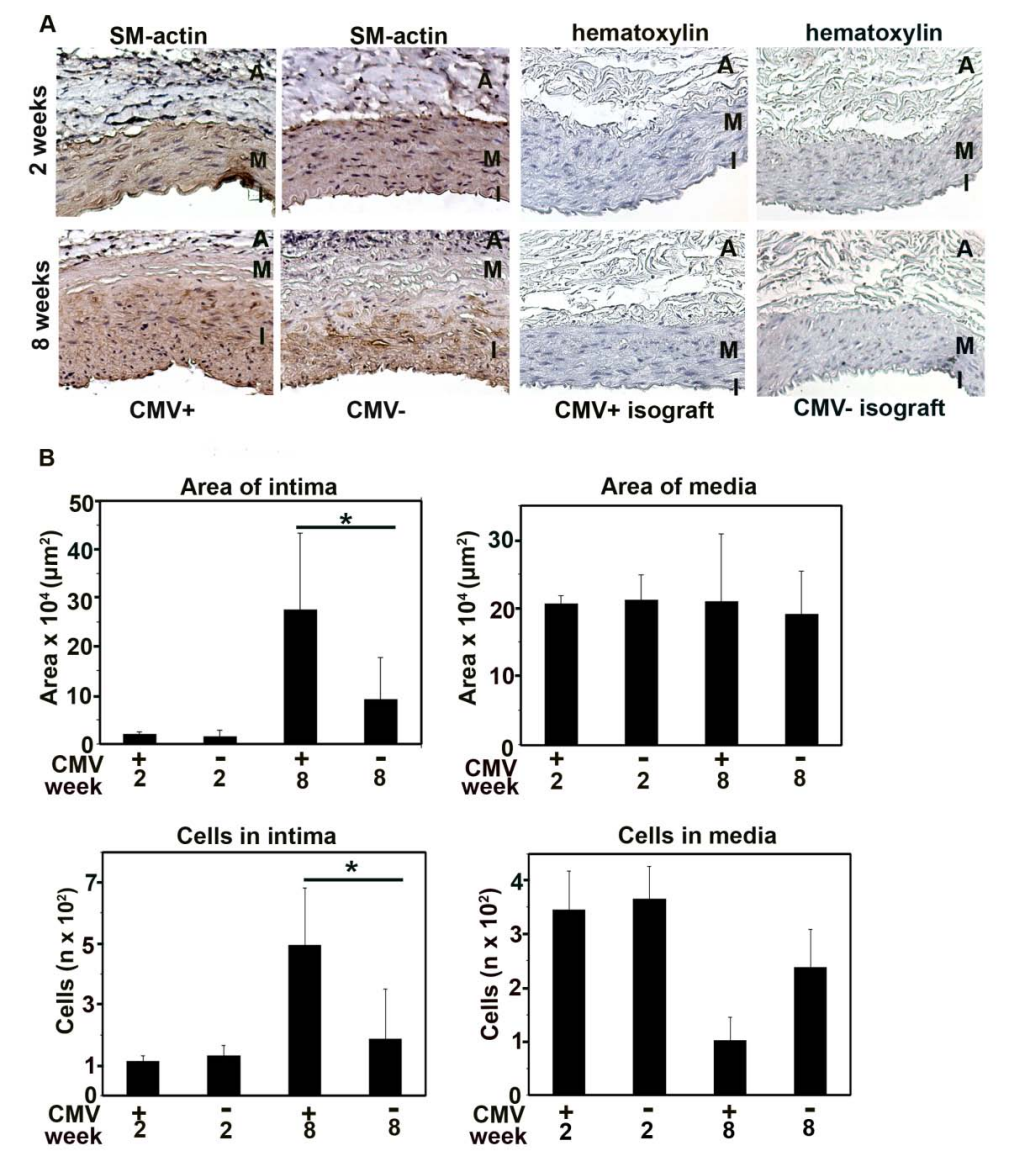
**RCMV infection of aortic allografts enhances intimal hyperplasia and contributes to vessel remodelling**. (A) The figure presents fragments of cross sections of RCMV-infected and uninfected allografts (controls) collected at 2 and 8 weeks after transplantation and stained for SM-*α*-actin (SM-actin) (Columns 1 and 2). Columns 3 and 4 show fragments of cross sections of the RCMV-infected and uninfected isograft controls at 2 and 8 weeks after transplantation to visualize that there was no intimal formation either in the infected or uninfected isografts. Blue, nuclei counterstained with hematoxylin. Brown, cells positive for SM-*α*-actin. I, intima, M, media, A, adventitia. (B) Bar charts show areas of intima and media presented as cross-sectional area (μm**^2^**) (upper panels) and the number of cells per cross section in the intima and media (lower panels) in RCMV-infected and uninfected allografts. At 2 weeks post transplantation the number of cells in the intima and media, and thickness of both layers were similar in infected and uninfected allografts. At 8 weeks, the intima was 3-fold thicker in infected ***vs. ***uninfected allografts and contained 2.5-fold more cells. The media of infected allografts had fewer cells at 8 week after transplantation. * p < 0.05 ***vs. ***control at 2 and 8 weeks, respectively.

To examine how RCMV infection affects arterial remodeling and intimal hyperplasia, we measured the cross-sectional area and circumference of the intima and media in the tissue sections and counted the number of cell nuclei counterstained with hematoxylin and cells of SM-α-actin phenotype in both media and intima (Figure 1A). We used the isograft control to confirm previous findings that allogenic response is necessary to initiate graft remodeling [36,39]. Neither uninfected nor infected isograft controls collected at 2 and 8 weeks post transplantation developed intimal hyperplasia (Figure 1A). At 8 weeks, the intimal cross-sectional area was threefold thicker in infected allografts (*p *< 0.05) (Figure 1B) and contained 2.5-fold more cells (*p *< 0.05) compared to uninfected allografts. Also the number of cells was increased in the intima of RCMV-infected allografts at 8 weeks after transplantation compared to uninfected allografts at the same time point. Our findings indicate that RCMV increased intimal hyperplasia in the allografts and contributed to vascular remodeling.

**Figure 2:**
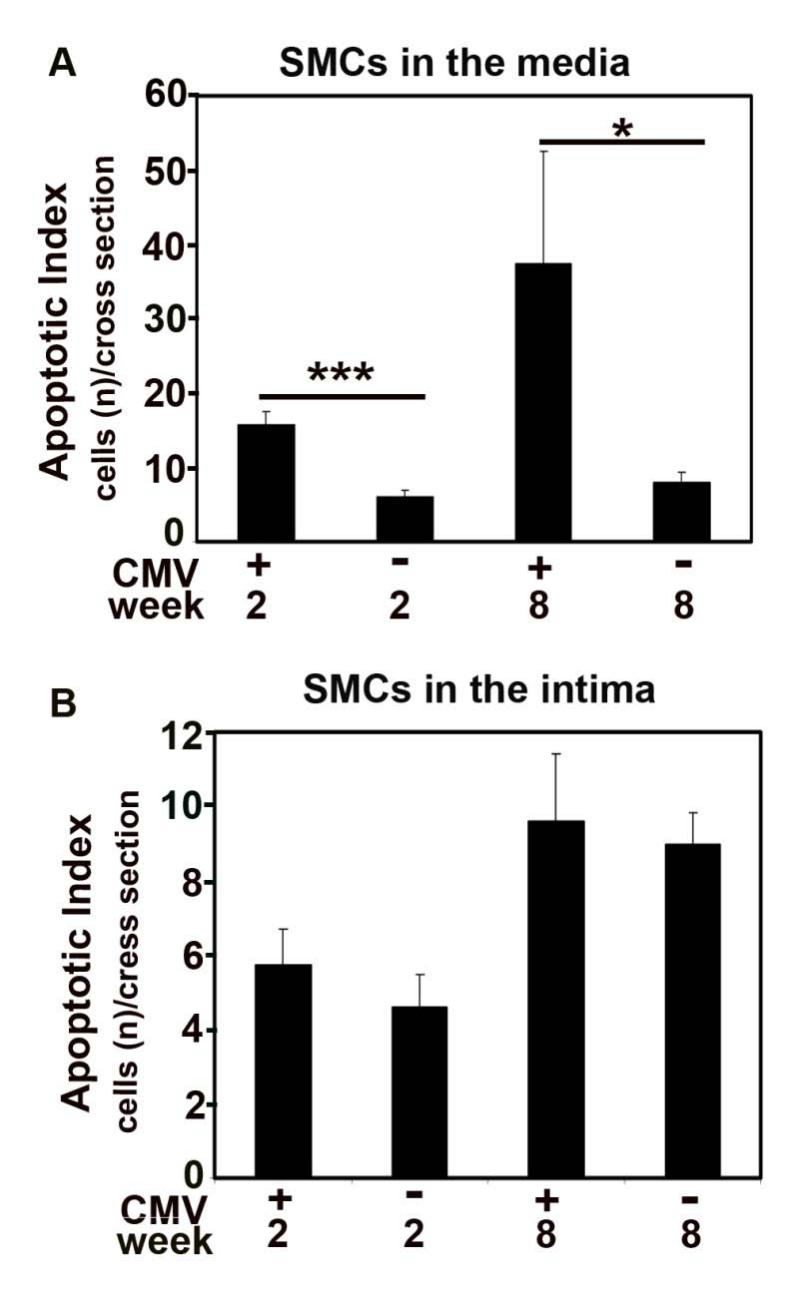
**RCMV induces apoptosis of SMCs in the media layer of the aortic allografts**. Bar charts show the Apoptotic Index of SMCs presented as the number of apoptotic SMCs per cross section in media (A) and intima (B) of infected and uninfected allografts (controls). Apoptotic SMCs were detected with double immunostaining for SM-*α*-actin and caspase 3. Both at 2 and 8 weeks after transplantation there was a significant increase of apoptotic SMCs in the media of infected allografts vs. uninfected controls. *p < 0.05 ***vs. ***control at 8 weeks. ***p < 0.001 ***vs. ***control at 2 weeks.

RCMV did not affect the media cross-sectional area, but infected grafts had fewer cells in the media (Figure 1B). To further examine whether this loss of medial cells could be attributed to an enhanced RCMV-induced apoptosis, we double stained RCMV-infected- and uninfected allografts for SM-α-actin and caspase 3 and found that apoptosis of SMCs was significantly increased in the media of infected allografts compared to uninfected controls both at 2 weeks (*p *< 0.001), and 8 weeks after transplantation (*p *< 0.05) (Figure 2).

**Figure 3:**
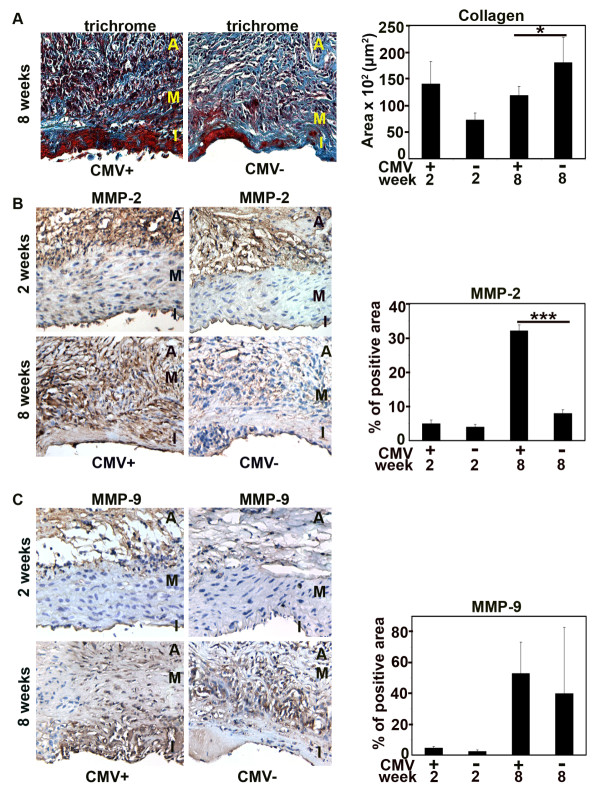
**RCMV increases expression of matrix metalloproteinases and decreases collagen deposition in the aortic allografts**. (A) Cross sections of RCMV-infected and uninfected allografts (controls) at 2 and 8 weeks post transplantation, stained with Masson's trichrome to identify collagen deposits. Blue, collagen; red, muscle fibers; black, nuclei; I, intima; M, media; A, adventitia. Bar chart shows the collagen deposits in the allografts of RCMV-infected and uninfected allografts at 2 and 8 weeks post transplantation presented as the size (μm**^2^**) of positive blue immunostained area per cross section. *p < 0.05 ***vs. ***uninfected controls 8 weeks after transplantation. At 8 weeks, infected allografts had significantly less collagen. (B) Cross sections of RCMV-infected and uninfected allografts (controls) stained to identify MMP-2. Brown indicates positivity for MMP-2. I, intima; M, media; A, adventitia. Bar chart shows percentage of positive areas for MMP-2 in infected and uninfected allografts at 2 and 8 weeks post transplantation determined as the percentage of positive area of the total cross section area. ***p < 0.001 ***vs. ***uninfected controls at 8 weeks. (C) Cross sections of RCMV-infected and uninfected allografts (controls) stained to identify MMP-9. Brown indicates positivity for MMP-9. I, intima; M, media; A, adventitia. Bar chart shows percentage of positive areas for MMP-9 in infected and uninfected allografts at 2 and 8 weeks post transplantation. Early after transplantation both MMP-2 and MMP-9 after confined mainly to the adventitia.

To further assess the effects of RCMV infection on the extracellular matrix (ECM) components, we examined collagen deposition (Figure 3A). At 2 weeks post transplantation, similar amounts of collagen were present in infected and uninfected allografts. At 8 weeks, RCMV-infected allografts had less collagen (*p *< 0.05) (Figure 3A), indicating that the virus directly or indirectly, affected collagen turnover in the allografts. Therefore, we determined the expression of two matrix metalloproteinases (MMPs) that are major regulators of collagen turnover in the vessel wall, MMP-2 and MMP-9 (Figure 3B and 3C). In infected grafts, MMP-2 expression was confined to the adventitia early after transplantation but was later detected in a diffuse pattern in the whole vessel wall and was significantly increased (*p *< 0.001) compared to uninfected allograft (Figure 3B). MMP-9 expression was not affected by RCMV infection (Figure 3C).

### RCMV infection of aortic allografts induces widespread inflammation in the intima and adventitia

**Figure 4:**
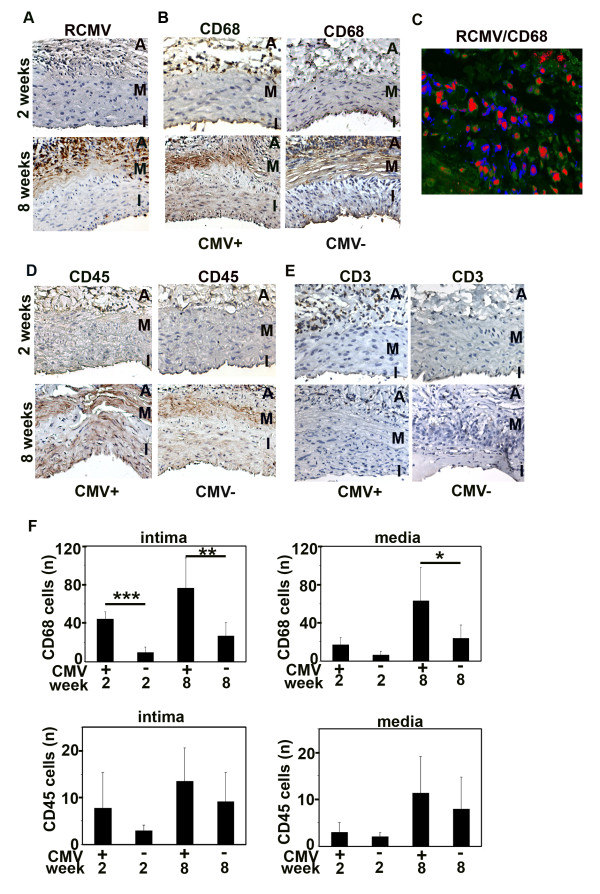
**RCMV induces inflammation in the aortic allografts with increased infiltration of macrophages**. (A) Cross sections of RCMV-infected allografts 2 and 8 weeks post transplantation stained to detect RCMV early/late antigen (R44-encoded protein). RCMV-infected cells were localized mainly in the adventitia and intima of the allograft. Brown, cells positive for RCMV. I, intima; M, media; A, adventitia. (B) Cross sections of RCMV-infected allografts and uninfected allografts (controls) at 2 and 8 weeks post transplantation stained for CD68-positive macrophages. Brown indicates positive staining for CD68 that appears mainly in the intima and adventitia. I, intima; M, media; A, adventitia. (C) Fragment of a cross section of RCMV-infected allograft double immunostained to detect RCMV-infected CD68-positive macrophages. 80% of infiltrating CD68-positive macrophages were RCMV infected. Green, CD68-positive macrophages; blue, RCMV; red, nuclei. (D) Cross sections of RCMV-infected allografts and uninfected controls at 2 and 8 weeks post transplantation stained for CD45-positive leukocytes. Brown indicates positive staining for CD45 that appears mainly in the intima and adventitia. I, intima; M, media; A, adventitia. (E) Cross sections of RCMV-infected allografts and uninfected controls at 2 and 8 weeks post transplantation stained for CD3-positive lymphocytes. Brown indicates positive staining for CD3 that appears mainly in the adventitia. I, intima; M, media; A, adventitia. (F) Bar charts show a number of CD68- and CD45-positive leukocytes in the media and intima of RCMV-infected and uninfected allografts (controls). Infiltration of CD68-positive macrophages into the intima was 4-fold higher and 2-fold higher in the media of infected allografts compared to uninfected controls. At 8 weeks, there was a massive infiltration of macrophages in the adventitia. ***p < 0.001 vs. uninfected control at 2 weeks in intima; **p < 0.01 vs. uninfected control at 8 weeks in intima; *p < 0.05 *vs*. uninfected control at 8 weeks in media.

Local RCMV infection resulted in a widespread inflammation of the intima and adventitia early after transplantation (Figure 4A). To further characterize the phenotypes of infiltrating cells in RCMV-infected rat aortic allografts, we determined the presence of CD68-positive macrophages, CD45-positive leukocytes and CD3-positive lymphocytes (Figure 4B, D, E). Interestingly, most of the infiltrating cells were CD68-positive macrophages (Figure 4B) and 80% of infiltrating macrophages were RCMV-infected at 8 weeks post transplantation (Figure 4C). CD3-positive lymphocytes were only few and mainly localized in the adventitia and subendothelial area of the allografts at 2 weeks post transplantation (Figure 4E). Moreover, infiltration of CD68-positive macrophages into the intima was fourfold higher in RCMV-infected allografts compared to uninfected controls, both 2 weeks (*p *< 0.001) and 8 weeks (*p *< 0.01) after transplantation (Figure 4F). In the media of infected allografts the number of CD68-positive macrophages was twofold higher at 8 weeks compared to uninfected controls (*p *< 0.05). In the adventitia, there was a massive infiltration of CD68-positive macrophages, with a higher extent in the infected allografts. We observed no significant effect of RCMV on infiltration of CD45-positive leukocytes to the allografts (Figure 4F). These findings indicate that RCMV induced a strong inflammatory response consisting of mainly CD68-positive macrophages in the allografts early and late after transplantation, which likely influenced allograft remodeling.

### RCMV induces MCP-1 production in aortic allografts and recruits cells from infected adventitia to the intima

Adventitial progenitor cells appear to be important in vessel remodeling [40-43]. Recently, we found that MCP-1 is a chemoattractant for vascular progenitor cells in cardiac allografts and that their recruitment to the allograft was increased by inflammation [11].

**Figure 5:**
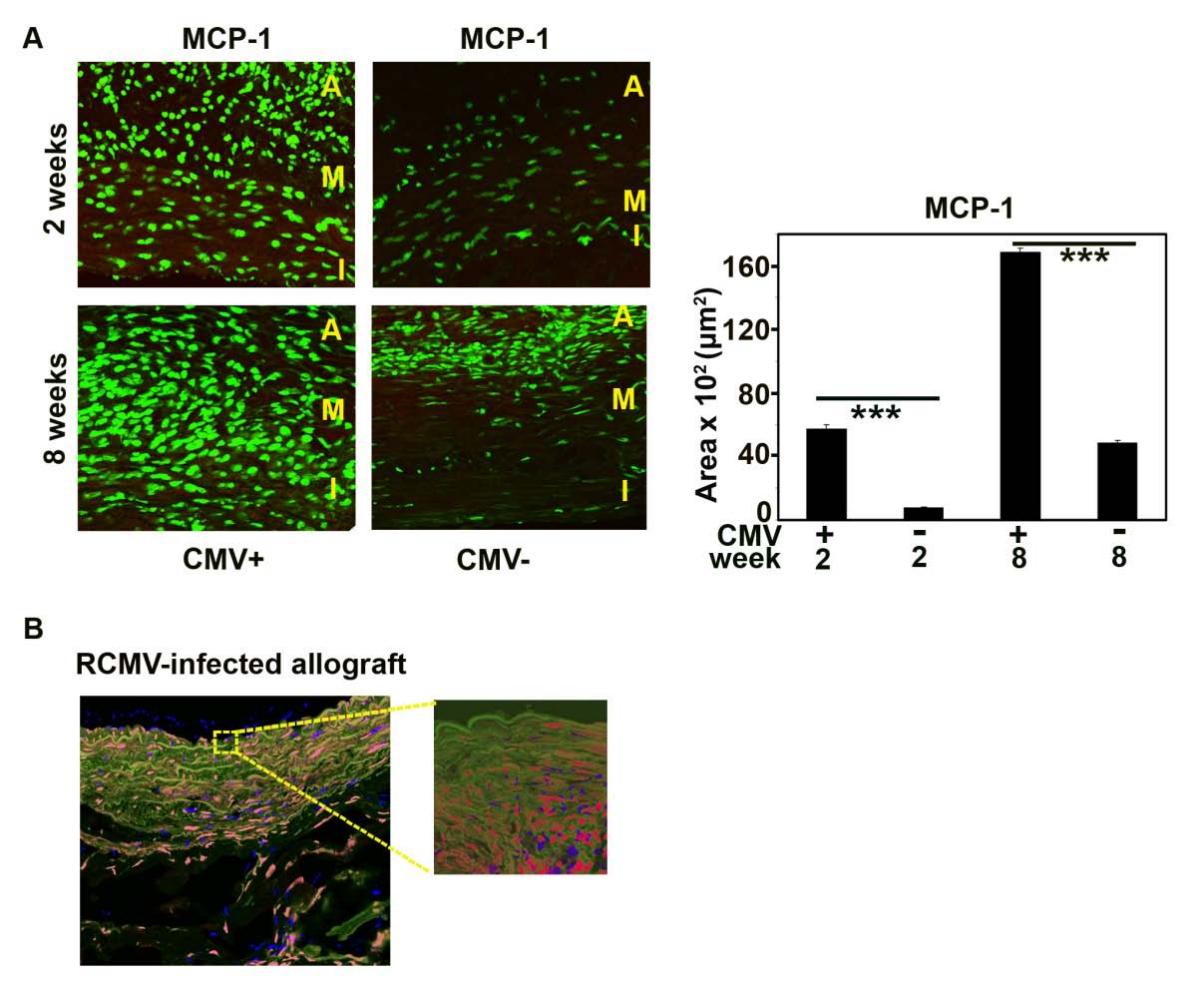
**RCMV increases expression of MCP-1 in the aortic allografts and cells from the infected adventitia migrate towards the intima**. (A) Cross sections of the RCMV-infected and uninfected allografts (controls) at 2 and 8 weeks post transplantation stained for MCP-1. Green, MCP-1. Bar chart shows MCP-1 expression in the allografts, presented as positive area of the cross section (μm**^2^**). Both at 2 and 8 weeks after transplantation MCP-1 expression was increased in infected allografts (7-fold at 2 weeks and 3.5-fold at 8 weeks post transplantation). ***p < 0.001 ***vs. ***uninfected control at 2 and 8 weeks, respectively. I, intima; M, media; A, adventitia. (B) Cross section of the aortic allograft with transplanted fluorescently labeled isogenic adventitial graft. Grafts were collected 2 weeks post adventitia transplantation. Picture shows labeled blue adventitial cells in the allograft intima. Green, SM-actin; Blue, migrated adventitial cells; Red, nuclei.

To further examine whether RCMV affected migration of adventitial cells in the rat allograft we first examined if MCP-1, which is a key chemokine responsible for migration of vascular progenitor cells, was altered by RCMV infection. At 2 weeks after transplantation, RCMV infection resulted in a sevenfold increase in the number of MCP-1 positive cells in the allografts (*p *< 0.001) and most of these cells were present in the adventitia (Figure 5A). At 8 weeks, RCMV-infection resulted in a 3.5-fold increase in the number of MCP-1 positive cells, which were present in all vascular layers (*p *< 0.001) (Figure 5A).

To further examine if cells from RCMV-infected adventitia migrated *in vivo *and thereby could contribute to intimal hyperplasia, we transplanted infected fluorescently labeled isogenic adventitia to the aortic allografts 4 weeks after transplantation, the time point when intimal hyperplasia is emerging [36]. At 2 weeks post adventitia transplantation, we detected fluorescently labeled cells in the intima (Figure 5B). Thus, RCMV induced MCP-1 expression *in vivo *and induced migration of adventitial cells to the intima.

## Discussion

CMV is thought to be a key pathogen involved in the pathogenesis of TV in human allografts. To evaluate the direct effect of CMV infection on vascular biology, we used a rat model in which aortic allografts were infected *ex vivo *with RCMV prior to transplantation. We chose 2 time points after transplantation to investigate the development of TV, 2 weeks that represented the early phase of TV in rats; and 8 weeks, when TV was fully developed. We found that RCMV influenced vascular remodeling by increased apoptosis of SM-α-actin positive cells in the media layer, decreased extracellular matrix deposits and increased intimal hyperplasia. Moreover, RCMV induced a strong infiltration of CD68-positive macrophages mainly in the adventitia and resulted in an increase of MCP-1 in the allograft, which resulted in migration of adventitial cells towards the intima that most likely also contributed to intimal hyperplasia.

Vessel stability is sustained by a balance between cellular proliferation and apoptosis, and the synthesis or degradation of extracellular matrix (ECM) components. Alterations in this balance have been shown to contribute to the development of vascular diseases. Previous studies suggested that HCMV infection negatively influenced coronary artery remodeling in the first year after heart transplantation [12]. Moreover, systemic RCMV infection stimulated arterial SMCs proliferation when vascular injury was induced in the rat model of restenosis [44], although in this particular study the ECM content in the injured artery was not determined.

In our study, we found that RCMV increased apoptosis of SM-α-actin positive cells in the media, which contributed to an earlier and enhanced destruction of the medial layer after transplantation compared to uninfected allografts. This destruction of the media layer led to further fibrosis and stimulation of intimal hyperplasia. RCMV increased proliferation of SM-α-actin positive cells in the intima resulting in intimal thickening in TV. Consistent with other studies, we observed no intimal formation in isogenic grafts (isografts) [22,36,45], even if they were infected with CMV [27,31,35], which suggests that allogenic response is necessary for CMV to further trigger cellular responses in TV.

Collagen is the major component of the ECM in the vessel wall and is crucial for vessel wall integrity. Here, we found that RCMV decreased the collagen content in the allografts late after transplantation. Moreover, expression of MMP-2, which regulates collagen turnover in the vessel wall and is thought to be altered in vascular diseases [46,47] was upregulated in infected allografts. These findings are in agreement with earlier report showing that the expression of proteins involved in MMP activation and ECM modification are induced in RCMV-infected cardiac allografts as TV develops [5]. These findings indicate that RCMV directly or by interacting with host immunity reduces the integrity of the artery wall and thereby increases tissue vulnerability to injury, inflammation and uncontrolled remodeling.

Previous studies showed that RCMV induces an early inflammatory response in the adventitia (perivasculitis) and the subendothelial space [26] in a rat aortic transplant model with systemic RCMV infection. However, the types of infected cells in the graft were not identified. In murine models of cardiac transplantation, mouse CMV infection resulted in chronic vascular rejection, intimal thickening, and vascular occlusion [48]. MCP-1 and its receptor (chemokine receptor 2) are though to drive the recruitment of inflammatory cells and cells with SM-α-actin phenotype to the sites of vascular injury in atherosclerosis and TV [49]. MCP-1 transcripts have been identified in RCMV-infected rat cardiac allografts affected with TV [5]. Recently we found that MCP-1 is a chemoattractant for progenitor cells migrating to cardiac allografts, which was enhanced by inflammation [11]. We also showed that antibodies to MCP-1 significantly reduced inflammation and accumulation of these cells in the graft.

In our study, we observed RCMV-dependent recruitment of CD68-positive macrophages. The infiltration of lymphocytes into the allograft was mainly confined to the adventitia and the subendothelial area early after transplantation. This pattern was also previously observed by us and other authors in the similar animal models of TV [36,39], as well as in the models of RCMV-infected rat aortic and cardiac allografts [26,50,51]. The lymphocytic infiltration was observed until 1 week post transplantation [26] but not thereafter and late inflammatory response consisted mainly of macrophages.

We also observed that MCP-1 is highly expressed in infected allografts in areas of the artery with high numbers of macrophages. This scenario may contribute to the pro-inflammatory state in the allograft, resulting in release of inflammatory cytokines and chemokines that accelerate the inflammatory process and sustain further viral replication [21] and vascular remodeling. Importantly, CMV also carries chemokines and chemokine receptor homologues that recruit and stimulate cellular infiltration, and thereby further potentiate the inflammatory response [52]. Furthermore, several studies have shown that infection with viruses can result in the up-regulation of the eicosanoid pathways [53,54]. A relationship between CMV and enhanced cyclooxygenase-2 (COX-2) expression has been identified both *in vitro *and *in vivo *[55,56]. COX-2 and prostaglandin pathways are up-regulated in CMV infected cells and this may be essential for efficient CMV replication [55]. Interestingly, in the CMV-infected SMCs, induced 5-Lipoxygenase expression and increased leukotriene B4 production may promote inflammation through induced infiltration and activation of leukocytes [17,57]. Thus, CMV infection of tissues may exacerbate inflammation and contribute to the observed pathology.

The vascular adventitia has been implied as a source of cells that contribute to intimal hyperplasia [41,43,58] and migrate in the vessel wall in order to localize in vascular lesions [59]. Here, we investigated whether cells derived from infected adventitia migrated to the intima and if migration of adventitial cells was altered by RCMV. We transplanted fluorescently labeled RCMV-infected adventitia to the allografts 4 weeks after the allograft transplantation and found that adventitial cells migrated to the intima and most likely contributed to intimal hyperplasia. Interestingly, we showed that RCMV infection significantly enhanced MCP-1 production in the allograft and thereby created the environment to support cellular migration. Our observation is consistent with our previous finding that expression of the virally encoded chemokine receptor chomologue, US28 in the presence of CC chemokines, including MCP-1, was sufficient to promote cellular migration [21]. Moreover, it suggests that the virus itself creates pro-inflammatory environment and is able to alter cellular processes involved in the development of TV.

## Conclusions

In summary, acute RCMV infection of allografts led to enhanced MCP-1 production and massive infiltration of macrophages, following transplantation, resulting in inflammation and vascular remodeling that contributed to more rapid and severe vessel narrowing. Our findings increase understanding of the CMV role in vessel remodeling and highlight the importance of managing CMV infections in transplant patients.

## Competing interests

The authors declare that they have no competing interests.

## Authors' contributions

MKG participated in the design of the study, performed the experiments, analyzed the data and wrote the manuscript. BK participated in the design of the study and performed the experiments. JS participated in the design of the study and performed the experiments. FS analyzed the data and helped to draft the manuscript. CSN participated in the study design, analyzed the data and assisted in writing the manuscript. PR participated in the study design and helped to analyze the data. All authors read and approved the final manuscript.
